# Colonic Adenocarcinoma at Advanced Stage in Adolescence: Report of 2 Cases

**DOI:** 10.1155/2020/1848367

**Published:** 2020-07-28

**Authors:** Divya S. Vundamati, Xiuxu Chen, Vivekanand Singh

**Affiliations:** ^1^University of Missouri School of Medicine, Kansas City, MO 64108, USA; ^2^Department of Pathology and Laboratory Medicine, University of Kansas Medical Center, 3901 Rainbow Blvd, Kansas City, KS 66160, USA; ^3^University of Texas Southwestern Medical Center, 5323 Harry Hines Blvd, Dallas, TX-75390, USA

## Abstract

**Background:**

Carcinoma of colon is rare in children and adolescents. The staging criteria of the carcinoma is the same as those for adults. However, the pathogenetic background in pediatric cases is different from adults and usually involves mismatch repair gene mutations or familial polyposis syndromes. *Case report*. We describe two adolescents diagnosed with advanced stage colon carcinoma and discuss the histological appearance, testing for mismatch repair genes and contrast- it with carcinoma occurring in the setting of familial polyposis syndrome.

**Conclusion:**

Colonic carcinoma occurring in pediatric patients should prompt a work-up for mismatch repair gene mutation status. Despite higher stage of presentation, some of the pediatric patients may respond favorably to chemotherapy and surgical resection.

## 1. Introduction

Colorectal cancer (CRC) is the fourth most common cancer in the United States, with an incidence rate of 38.0 and death rate of 14.0 per 100,000 people in 2016 [1]. However, adenocarcinoma of colon is very rare in children and adolescents. Only 1.4 new cases per 100,000 people were reported in age group 15-19 years in 2016 and even lower in age younger than 15 years [1]. Lynch syndrome and polyposis syndromes such as familial adenomatous polyposis (FAP) syndrome and MUTYH-associated polyposis syndrome are the usual settings in which CRC occurs in young patients. In this study, we report two cases of colon adenocarcinoma with peritoneal metastasis that were diagnosed in adolescents and discuss their clinicopathological features.

## 2. Case Summaries

### 2.1. Clinical History 1

**Figure 1:**
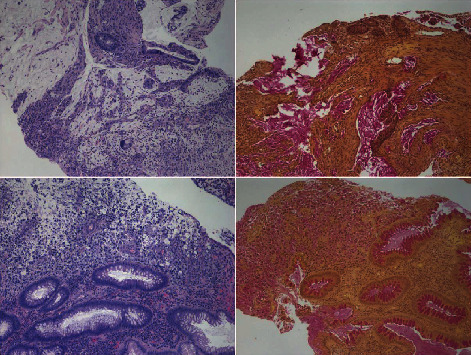
Top row shows H&E-stained image (×100 magnification) of colon carcinoma from case 1 in the left panel and mucicarmine stain staining mucin (red) in the right panel. Bottom row shows H&E-stained image (×100 magnification) of colon carcinoma from case 2 in the left panel and mucicarmine stain staining mucin (red) in the right panel.

A 13-year-old male presented with vomiting, weight loss, and abdominal discomfort. His family history was not significant for cancer. A CT scan of the abdomen and pelvis revealed a large amount of free fluid in the peritoneal cavity with peripheral enhancement. There was circumferential, mural thickening of the cecum and proximal ascending colon. An exploratory laparoscopy showed diffuse metastatic deposits covering all peritoneal surfaces and almost totally replacing the omentum. There was no significant involvement of the liver; however, there was metastasis to the falciform ligament. A biopsy of the tumor removed from the omentum showed high-grade, mucinous adenocarcinoma with many signet ring cells (Figure 1). The tumor cells were positive for epithelial markers: cytokeratin 20 and MOC-31. Immunohistochemical staining for mismatch repair proteins showed strong expression for MLH1 and MSH2 in 80% tumor cells while MSH6 showed weak expression in only 10% cells. There was no genetic testing performed to exclude microsatellite instability, and thus, Lynch syndrome while suspected could not be unequivocally confirmed. The tumor due to extensive peritoneal surface involvement was designated as stage 4. The patient received chemotherapy with folinic acid, 5-fluorouracil, and oxaliplatin regimen (FOLFOX). After 3 cycles of chemotherapy, he developed multiple side effects. The family opted for hospice care, and he soon expired.

### 2.2. Clinical History 2

An 18-year-old female presented with right lower abdominal pain and a two-month history of change in bowel habits including occasional blood in her stool. A colonoscopy was performed, and the rectosigmoid mucosal biopsies showed invasive, mucinous adenocarcinoma with many signet ring cells (Figure 1). Immunohistochemistry staining showed tumor cells positive for epithelial markers: CK20 and pancytokeratin. Immunohistochemical staining for mismatch repair proteins showed normal expression of MLH1, MSH2, MSH6, and PMS2. Her tumor was also tested at a reference laboratory for microsatellite instability by next generation sequencing technique and was negative for mutations in the MLH1, MSH2, MSH6, PMS2, and BRAF genes. A staging laparoscopy revealed extensive peritoneal surface involvement and metastases to ovaries. She received chemotherapy with FOLFOX and irinotecan and then underwent radical surgery with resection of colon, uterus, tubes, and ovaries. The resected colon showed transmurally invasive tumor (T4) and metastases to one pericolic lymph node, uterine serosa, and left ovary. The last follow-up of patient was at 1 year postsurgery, and she is free of tumor.

## 3. Discussion

In this study, we report two cases of colonic adenocarcinoma with wide spread metastases of peritoneum and pelvis in adolescent patients. Both of them presented with abdominal discomfort or pain, and histology revealed mucinous adenocarcinoma with the presence of signet ring cells. An earlier study has reported that colonic adenocarcinoma in children and adolescents although rare tends to have a higher stage, more often in the right colon, and has a high-grade histology of poorly differentiated adenocarcinoma or signet ring cell carcinoma [2]. Both of our patients had colonic carcinoma that had high-grade features with histological appearance of poorly differentiated adenocarcinoma with increased signet ring cells. Both cases did not meet the diagnostic criteria of signet ring cell carcinoma, which requires the presence of 50% area of signet ring cells.

Lynch syndrome, also referred to as Hereditary Nonpolyposis Colorectal Cancer (HNPCC), is caused by germline mutations in genes involving DNA mismatch repair (MMR), including MLH1, MSH2, MSH6, PMS1, and PMS2. Mutation of these genes result in increased mutation in genome by reduced fidelity of genetic information during mitosis. Therefore, these patients have an increased frequency of microsatellite instability in their genome [3]. DNA mismatch repair testing in colon cancers is currently done by polymerase chain reaction (PCR) for five microsatellite loci (≥2 out 5 considered positive) and/or immunohistochemistry for four main MMR proteins including MLH1, MSH2, MSH6, and PMS2 [4]. Both of our cases were analyzed by the latter method by reference laboratories. Of the two patients reported here, case 1 demonstrated loss of MSH6 expression. Historically, patients with MMR mutations tend to have colon carcinoma in the cecum or right colon and have higher grade, such as that seen in case 1. MMR mutations cause familial colorectal cancer to become resistant to 5-fluorouracil therapy so fluoropyrimidine-based chemotherapy is less beneficial for patients with MMR mutations [5]. On the other hand, tumors with MMR mutations may respond better to immune checkpoint inhibitors [6]. More effective adjuvant chemotherapy with less resistance is required for patients with MMR tumors and high-risk features such as stage T4 disease.

In contrast to HNPCC, the carcinoma occurring in patients with polyposis syndromes tends to occur beyond the age of adolescence. The majority of children with APC germline mutations (FAP syndrome) are asymptomatic and undergo evaluation based on their family history. Children with FAP develop hundreds to thousands of adenomatous polyps mainly occurring in the colon and rectum. One study suggested that the optimal cut-off age for predicting the development of cancer in individuals with profuse type of FAP is 27 years [7]. One case report of a 16-year-old boy with FAP who developed advanced rectal carcinoma [8] noted that the boy had attenuated form of FAP. Histologically, carcinoma in the context of FAP develops mostly in the setting of a preexisting adenoma and tends to be moderate to well-differentiated. Interestingly, the carcinoma in profuse or classic FAP is left-sided whereas in attenuated FAP, it is more frequently in the distal colon or rectum. Risk of colon carcinoma occurring in individuals with MUTYH mutations tends to be more frequent when mutations are biallelic [9]. The polyps in patients with MUTYH mutations show sessile and serrated adenoma, and the histology of carcinoma tends to be mucinous adenocarcinoma with left-sided preponderance and increased presence of tumor-infiltrating lymphocytes [10]. Most patients develop carcinoma in adulthood, and childhood cases with MUTYH mutations were not reported in the literature.

## 4. Conclusion

In summary, carcinoma of colon occurring in adolescents is rare, and when it occurs, the possibility of underlying HNPCC syndrome should be considered. Very rarely, the carcinoma could be sporadic. In this report, we describe a case each of carcinoma occurring in HNPCC background and sporadically. Both patients received treatment protocols similar to adults of corresponding stage of colorectal carcinoma.
